# Intravascular large B-cell lymphoma appearance on dual-energy computed tomography: a case report

**DOI:** 10.1186/s12890-023-02420-9

**Published:** 2023-04-18

**Authors:** Daisuke Yamada, Ryosuke Imai, Masaki Matsusako, Yasuyuki Kurihara

**Affiliations:** 1 Department of Radiology, St. Luke’s International Hospital, 9-1 Akashi-Cho, Chuo-Ku, Tokyo, 104-8560 Japan; 2 Department of Pulmonary Medicine, Thoracic Center, St. Luke’s International Hospital, 9-1 Akashi-Cho, Chuo-Ku, Tokyo, 104-8560 Japan

**Keywords:** IVLBCL, Dual-energy CT, Pulmonary hypoperfusion, Hypoxemia, Iodine mapping

## Abstract

**Background:**

Intravascular large B-cell lymphoma (IVLBCL) is the proliferation of neoplastic B lymphocytes in the vascular space. Since conventional computed tomography (CT) shows nonspecific findings, differentiation between IVLBCL and other lung diseases, such as diffuse interstitial lung disease, is difficult.

**Case presentation:**

A 73-year-old man presented with dyspnea and hypoxemia. Laboratory findings showed an increased lactate dehydrogenase level of 1690 U/L (normal: 130–235 U/L) and soluble interleukin-2 receptor level of 1140 U/mL (normal: 157–474U/mL). Dual-energy CT iodine mapping showed a significant symmetrical decrease in iodine distribution in the upper lungs, suggesting an unusual distribution of pulmonary hypoperfusion. Therefore, IVLBCL was suspected. A random skin biopsy confirmed the diagnosis of IVLBCL. Due to the severity of the disease, lung biopsy was averted. After admission to the hospital, high-dose methotrexate was administered for central nervous system involvement, due to findings of suspected intracranial infiltration on a brain magnetic resonance imaging and elevated cell counts on lumbar puncture. Subsequently, oxygen demand improved, and rituximab along with cyclophosphamide, doxorubicin, vincristine, and prednisone was added to the patient’s regime. Eventually, oxygen administration was terminated, the patient’s general condition improved, and the patient was discharged after 47 days of hospitalization.

**Conclusions:**

Since the diagnosis of IVLBCL depends on whether it is possible to suspect IVLBCL, the finding of decreased iodine perfusion demonstrated on dual-energy CT is considered important information for diagnosis. An immediate diagnosis of IVLBCL is needed to avoid rapid disease progression and introduce early treatment for a favorable prognosis. In this case, unique pulmonary hypoperfusion demonstrated by dual-energy CT promoted early diagnosis of IVLBCL.

## Background

Intravascular large B-cell lymphoma (IVLBCL) of lung is a rare type of extranodal large B-cell lymphoma (LBCL) characterized by the selective growth of lymphoma cells within the lumina of vessels, particularly within capillaries, with sparing of larger arteries and veins. Conventional CT scans only present nonspecific imaging findings for IVLBCL. However, we present a case in which the newly developed dual-energy CT provided support for the diagnosis of IVLBCL.

## Case presentation

**Fig. 1:**
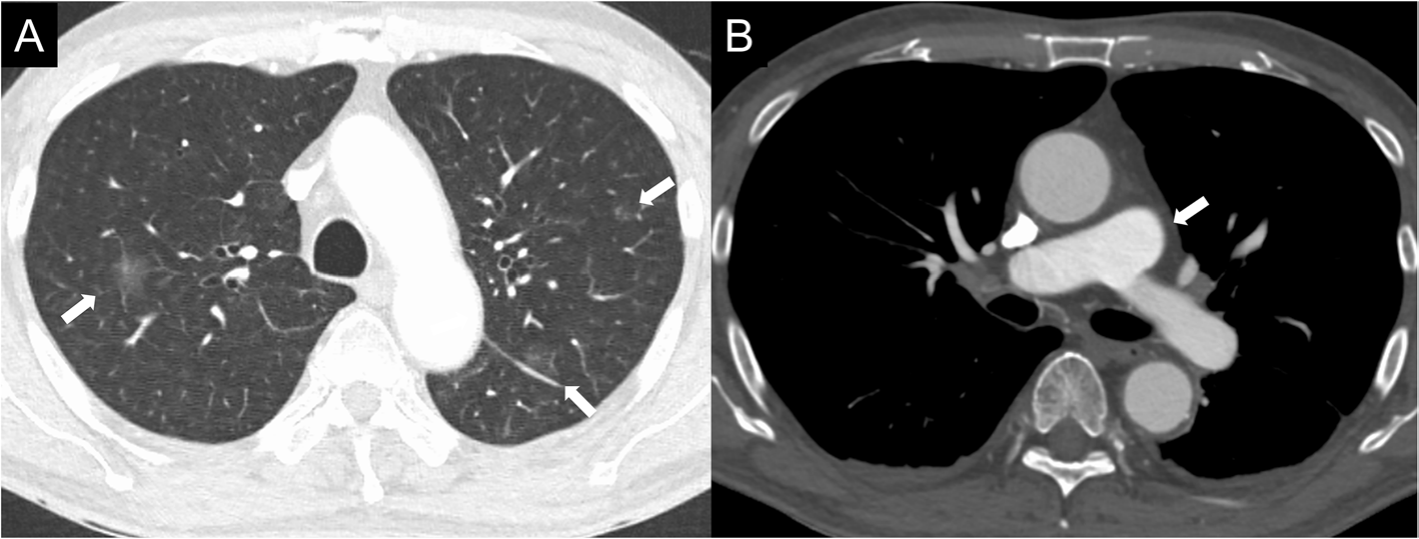
Chest computed tomography images. **A** Chest computed tomography (CT) shows patchy ground-glass opacities predominantly in both upper lobes of the bilateral lungs (arrows). **B** The pulmonary artery phase of the CT shows no dilatation of the main pulmonary artery trunk diameter and no contrast defects within the pulmonary arteries (arrow)

**Fig. 2:**
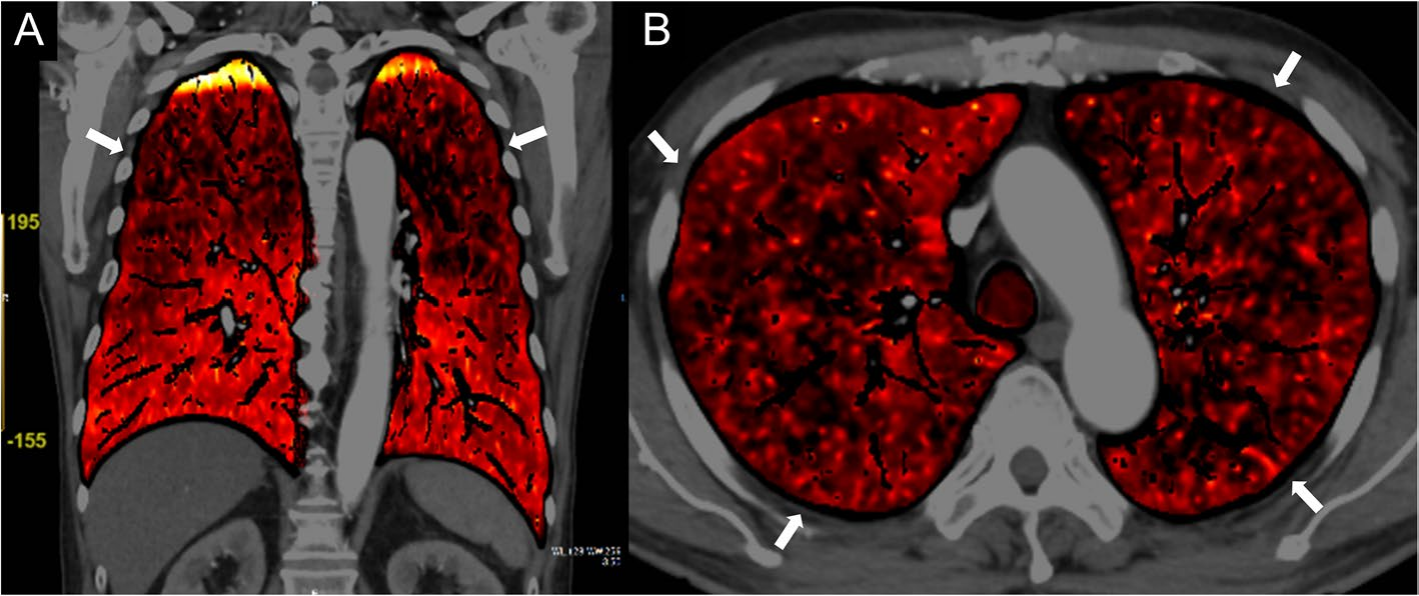
Dual-energy computed tomography images. **A**, **B** A color-coded iodine mapping image from the dual-energy CT shows decreased pulmonary perfusion in the upper lungs (arrows). The symmetrical, upper-lung dominant hypoperfusion was unusual for pulmonary thromboembolism and probably resulted from small vessel obstructive disease due to intravascular large B-cell lymphoma. The small yellow regions observed in the bilateral apices are attributable to hard beam artifact and do not imply high iodine concentration

A 73-year-old man presented with dyspnea and hypoxemia. The patient was admitted to our hospital because he had been experiencing dyspnea for the past 2 weeks at rest as well as during exertion. He had no history of smoking. On physical examination, the patient had a body temperature of 36.7 °C, blood pressure of 151/89 mmHg, heart rate of 122 beats/min, respiratory rate of 21 breaths/min, and oxygen saturation of the peripheral artery of 88% (room air). On auscultation, no heart murmur was heard and lung sounds were clear. Laboratory findings showed an increased lactate dehydrogenase (LDH) level of 1690 U/L (normal: 130–235 U/L) and soluble interleukin-2 receptor (sIL-2R) level of 1140 U/mL (normal: 157–474U/mL). We initially suspected pulmonary artery thromboembolism. Therefore, we performed a dual-energy Computed tomography (CT) scan, including the pulmonary artery phase. CT showed patchy ground-glass opacities predominantly in both upper lobes of the bilateral lungs (Fig. 1A). The pulmonary artery phase of CT showed no dilatation of the main pulmonary artery trunk diameter and no contrast defects within the pulmonary arteries (Fig. 1B). Dual-energy CT iodine mapping showed a significant symmetrical decrease in iodine distribution in the upper lungs, suggesting an unusual distribution of pulmonary hypoperfusion (Fig. 2).

**Fig. 3:**
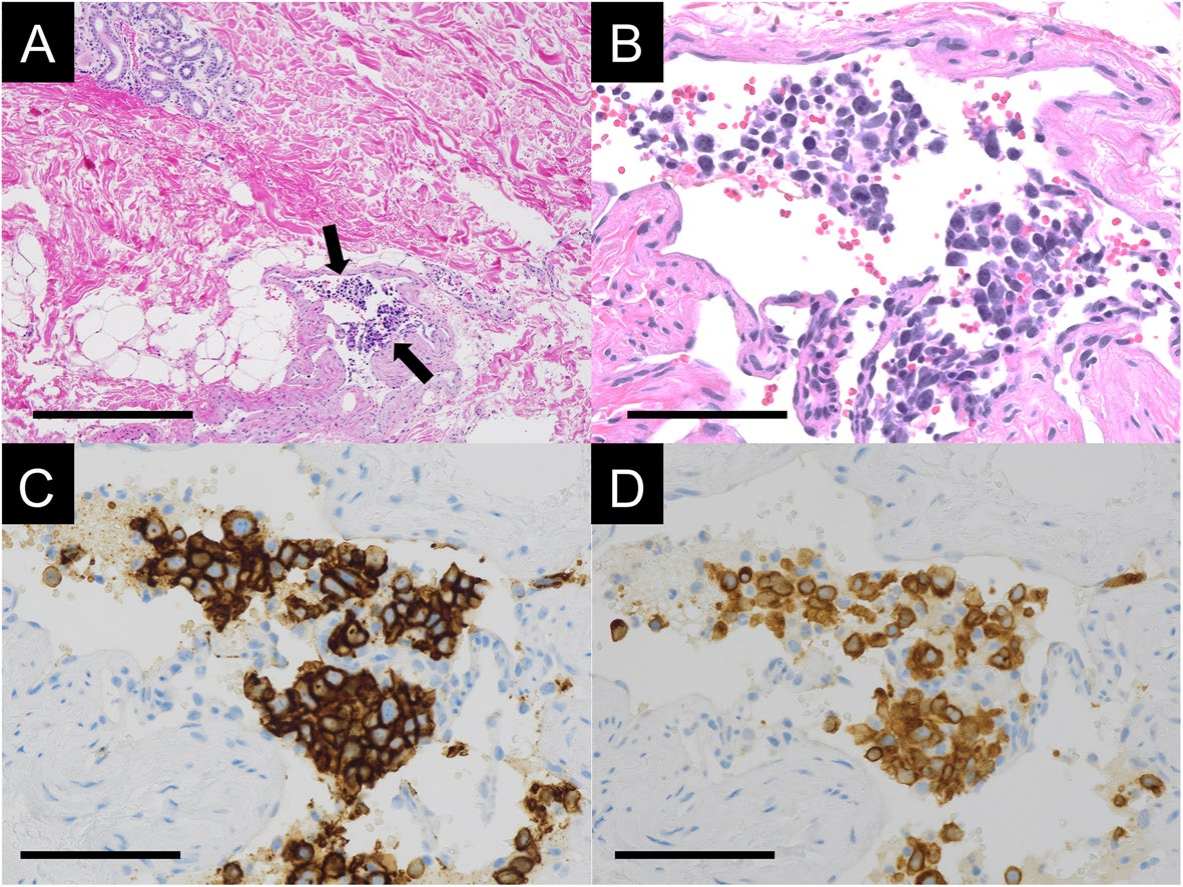
Histological images of a random skin biopsy specimen. **A**, **B** Hematoxylin and eosin stain (A: scale bar, 400 μm, B: scale bar, 200 μm) reveal a blood vessel filled with atypical lymphocytes (arrows). **C** Immunostaining shows CD20-positive lymphocytes within the vessel (scale bar, 100 μm). **D** Immunostaining shows CD79a-positive lymphocytes within the vessel (scale bar, 100 μm)

We suspected intravascular large B-cell lymphoma (IVLBCL) based on the patient’s history of dyspnea for the past 2 weeks with no history of cancer, decreased peripheral perfusion of the lung parenchyma on CT, and elevated LDH and sIL-2R levels on laboratory findings. Three days after admission, a random skin biopsy was performed. Atypical cells were present from the dermis to subcutaneous vessels, leading to the suspicion of lymphoma. Immunostaining showed that the atypical cells were CD5 + , CD20 + , CD79a + , CD3-, and CD30- (Fig. 3); therefore, the diagnosis of IVLBCL was confirmed. Due to the severity of the disease, lung biopsy was averted.

After admission to the hospital, high-dose methotrexate was administered for central nervous system involvement based on the findings of suspected intracranial infiltration on a brain magnetic resonance imaging and elevated cell counts on lumbar puncture. Subsequently, oxygen demand improved, and rituximab along with cyclophosphamide, doxorubicin, vincristine, and prednisone was added to the patient’s regime. Eventually, oxygen administration was terminated, the patient’s general condition improved, and the patient was discharged after 47 days of hospitalization.

## Discussion and conclusions

IVLBCL is the proliferation of neoplastic B lymphocytes in the vascular space. It is characterized by vascular obstruction of various organs. The diagnosis of IVLBCL before death is often difficult because of the diverse clinical presentation. When the lungs are involved, patients develop dyspnea, hypoxemia, and rarely pulmonary hypertension [1]. Since conventional CT shows nonspecific findings, differentiation of the lesions from other lung diseases, such as diffuse interstitial lung disease, is difficult [2]. Pulmonary perfusion scintigraphy shows diffuse peripheral circulatory disturbances in cases of IVLBCL [3]. However, perfusion scintigraphy is difficult to perform promptly due to the time required for nuclide preparation. Iodine mapping by dual-energy CT is useful for evaluation of pulmonary perfusion defects, with good agreement with lung perfusion scintigraphy [4]. This technique produces an iodine-specific image based on two CT datasets acquired with different X-ray spectra. On a color-coded iodine mapping image, the brighter-colored areas indicate a higher concentration of iodine (normal perfusion), and the darker-colored areas indicate a lower concentration of iodine (hypoperfusion area). Diffuse circulatory disturbances in the peripheral lung parenchyma of IVLBCL can be demonstrated by dual-energy CT as decreased iodine perfusion. Dual-energy CT is a simple and rapid procedure. Diffuse hypoperfusion of iodine on dual-energy CT can also be seen in other diseases that diffusely involve peripheral blood vessels in the lung parenchyma, such as pulmonary tumor thrombotic microangiopathy [5]. Nevertheless, since the diagnosis of IVLBCL depends on the suspicion of IVLBCL, the finding of decreased iodine perfusion demonstrated on dual-energy CT is considered important information for diagnosis. An immediate diagnosis of IVLBCL is needed to avoid rapid disease progression and introduce early treatment for a favorable prognosis. In this case, unique pulmonary hypoperfusion demonstrated by dual-energy CT promoted early diagnosis of IVLBCL.

## Data Availability

Data sharing is not applicable to this report as no datasets were generated or analyzed during the current study.

## Abbreviations

IVLBCL: Intravascular large B-cell lymphoma
CT: Computed tomography
LDH: Lactate dehydrogenase
sIL-2R: Soluble interleukin-2 receptor
